# HydroMark Clip-Guided Cryoablation of a <3-mm Breast Cancer at the Lower Limit of Sonographic Conspicuity in a High-Risk Surgical Patient

**DOI:** 10.7759/cureus.114521

**Published:** 2026-08-14

**Authors:** Vinicius Fornazari, Grit A Adler, Neal Hall, Yusuf Alzoubi, Renato Abu Hana

**Affiliations:** 1 Radiology, University of Florida College of Medicine, Jacksonville, USA; 2 Internal Medicine, HCA Florida Blake Hospital, Bradenton, USA

**Keywords:** biopsy clip localization, breast cryoablation, early-stage breast cancer, fiducial marker, hydrodissection, hydromark clip, image-guided cryoablation, invasive ductal carcinoma, percutaneous ablation, ultrasound-guided intervention

## Abstract

Breast cryoablation is emerging as a minimally invasive local treatment for carefully selected patients with low-risk, early-stage breast cancer. We report the case of a 73-year-old woman with a 3-mm, Nottingham grade 1, estrogen receptor-positive, progesterone receptor-positive, human epidermal growth factor receptor 2 (HER2)-negative invasive ductal carcinoma (cT1a cN0, stage IA) who was considered a poor surgical candidate because of severe pulmonary disease, chronic liver disease, active tobacco and alcohol use, peripheral arterial disease, and ongoing antiplatelet therapy. Following ultrasound-guided core needle biopsy, the tumor became poorly conspicuous, while the sonographically visible HydroMark clip (Devicor Medical Products, Inc., Cincinnati, OH) remained centered within the biopsy site. The clip was therefore used as the primary landmark for probe positioning during percutaneous ultrasound-guided cryoablation. Real-time ultrasonography confirmed complete ice-ball coverage of the clip-centered target, and hydrodissection maintained a protective plane between the ablation zone and the skin. The procedure was completed without complication. At the two-month follow-up, contrast-enhanced breast MRI and targeted ultrasound demonstrated expected post-ablation changes without suspicious residual enhancement or a residual mass. This case demonstrates that a HydroMark clip can serve as a reliable sonographic landmark for target localization and probe positioning when a small tumor becomes poorly conspicuous after biopsy. Although early imaging findings were favorable, longer-term follow-up is needed to confirm durable local tumor control.

## Introduction

Breast cancer management has progressively shifted toward less invasive, breast-conserving strategies. Cryoablation, which destroys a tumor by freezing it through a thin percutaneous probe rather than surgical excision, is particularly attractive for patients who cannot safely undergo surgery, because it is performed through a small skin puncture without general anesthesia or a surgical wound. It is among the most extensively studied percutaneous ablative options for selected patients with early-stage invasive ductal carcinoma (IDC). In the ACOSOG Z1072 trial, complete tumor ablation was achieved in most treated cancers, and in all tumors smaller than 1 cm, although patients still underwent surgical excision afterward to confirm the result pathologically [1]. The prospective ICE3 registry further supported cryoablation without excision in highly selected older women with small, hormone receptor-positive, human epidermal growth factor receptor 2 (HER2)-negative tumors, reporting a low rate of tumor recurrence in the treated breast at five years [2].

Despite this growing evidence base, medically complex and surgically high-risk patients remain underrepresented, even though they may derive substantial benefit from a minimally invasive outpatient procedure. An additional problem is technical: cryoablation depends on seeing the target on ultrasound during treatment, yet a very small tumor can become difficult or impossible to see after a core needle biopsy, which removes or disrupts part of the lesion. When the tumor is no longer visible, it cannot be reliably targeted. A biopsy clip - a tiny permanent marker deployed at the biopsy site to indicate the tumor's location - can address this, because it remains visible on ultrasound and serves as a fiducial landmark, a fixed reference point that guides probe placement even after the tumor itself has become inconspicuous. We report ultrasound-guided percutaneous cryoablation of a 3-mm IDC in a high-risk surgical patient, using a HydroMark clip (Devicor Medical Products, Inc., Cincinnati, OH) placed at diagnostic biopsy as the primary sonographic landmark for target localization and probe positioning.

## Case presentation

A 73-year-old woman with multiple comorbidities presented after a right breast lesion was detected on routine screening mammography. She denied breast-related symptoms, including a palpable mass, nipple inversion, nipple discharge, or skin changes. Ultrasound-guided core-needle biopsy confirmed IDC, Nottingham grade 1, estrogen receptor (ER)-positive, progesterone receptor (PR)-positive, HER2-negative, with a low Ki-67 proliferative index (<10%). Diagnostic breast imaging and clinical evaluation established a right breast cT1a cN0, stage IA tumor measuring approximately 3 mm in greatest dimension.

Her medical history was notable for laryngeal and vulvar carcinomas previously treated with chemotherapy and radiation, as well as hypertension, hyperlipidemia, peripheral arterial disease, chronic obstructive pulmonary disease, chronic hepatitis B and C, active tobacco use of approximately one pack per day, and heavy daily alcohol consumption of six to seven beers. She was receiving clopidogrel for peripheral arterial disease.

The case was reviewed in a multidisciplinary setting with the patient and her caregiver. Lumpectomy, with consideration of sentinel lymph node biopsy omission given the small tumor size, clinically node-negative status, favorable biology, age, and comorbidity burden, was discussed by the surgical team. Because of severe pulmonary disease, chronic liver disease, active smoking and heavy alcohol use, prior neck and pelvic radiation, and ongoing antiplatelet therapy, she was considered a poor surgical candidate. After informed consent and shared decision-making, she elected percutaneous cryoablation as a minimally invasive local treatment. The HydroMark clip placed at core needle biopsy was used as a fiducial marker for procedural localization and subsequent imaging surveillance.

Imaging work-up

**Figure 1:**
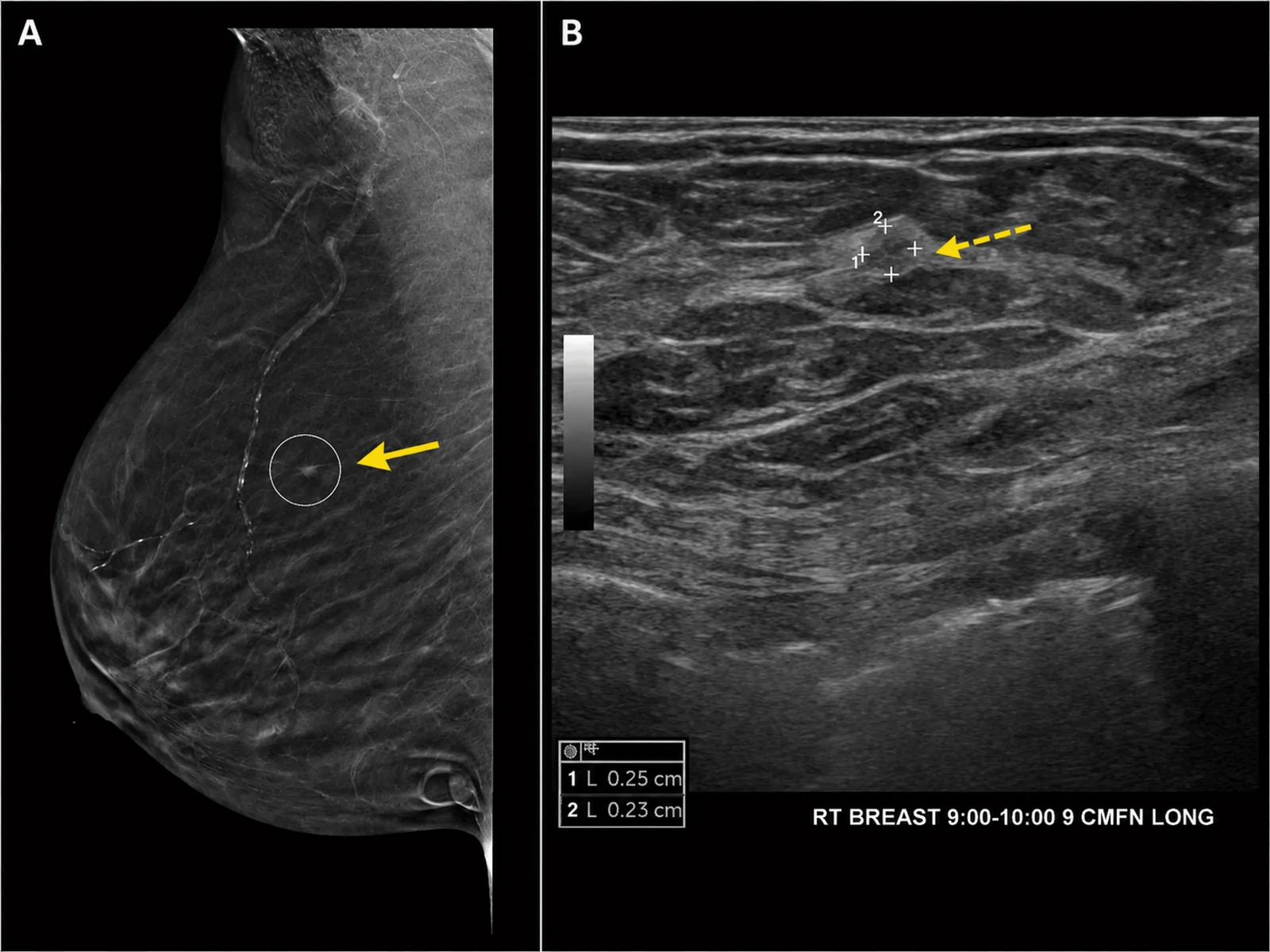
Preprocedural imaging. (A) Right mediolateral oblique mammographic view demonstrates a small abnormality in the upper outer right breast (arrow). (B) Targeted ultrasound demonstrates a 2.5 × 2.3-mm hypoechoic mass at the 9-10 o'clock position, 9 cm from the nipple (dashed arrow).

Preprocedural imaging (Figures 1A-1B) included diagnostic mammography and targeted breast ultrasonography. Mammography demonstrated scattered fibroglandular density with a persistent approximately 3-mm abnormality in the upper-outer quadrant of the right breast, 9 cm from the nipple. Targeted ultrasound identified a 2.5 × 2.3-mm hypoechoic mass with indistinct margins at the 9-10 o'clock position, 9 cm from the nipple, without suspicious ipsilateral axillary lymphadenopathy. The lesion was classified as BI-RADS 4 (Breast Imaging Reporting and Data System category 4), and tissue sampling was recommended.

**Figure 2:**
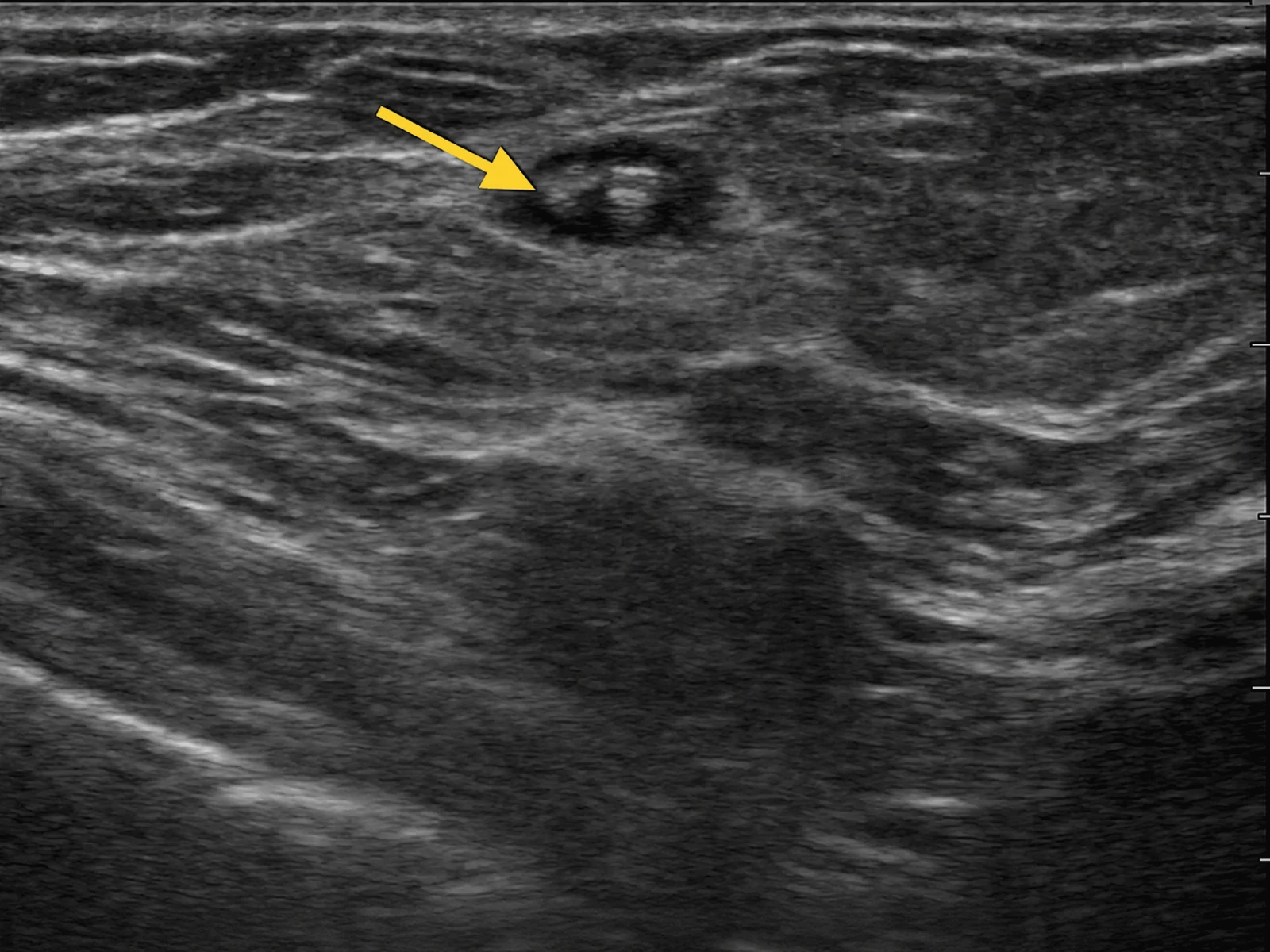
Post-biopsy targeted ultrasound. Post-biopsy targeted ultrasound demonstrates the echogenic HydroMark clip centered within the biopsy site (arrow). The original mass is poorly conspicuous following core-needle sampling.

Ultrasound-guided core-needle biopsy was performed using a 12-gauge Bard Marquee needle with multiple passes through the target. A HydroMark clip was deployed at the biopsy site, and post-biopsy ultrasound (Figure 2) confirmed that the marker remained centered within the mammographic target without clinically relevant migration. Histopathologic analysis revealed well-differentiated IDC with tubular features, Nottingham Grade 1 (tubule formation 1, nuclear pleomorphism 1, mitotic count 1), without lymphovascular invasion. Immunostaining for p63 was negative, and imaging-pathology concordance was confirmed. Because the tumor measured only approximately 3 mm, it became poorly conspicuous following biopsy; the sonographically visible clip therefore served as the critical landmark for cryoablation planning and target localization. Clinicians attempting to reproduce this approach in a similarly small lesion should anticipate that multi-pass core sampling may remove or obscure the original hypoechoic mass, so the target visible at the time of ablation is typically not the tumor itself but the echogenic HydroMark clip centered within the post-biopsy hematoma bed. In this setting the clip functions as a direct sonographic surrogate for the now-inconspicuous tumor. 

Procedure description

Preprocedural Planning

The patient was evaluated and considered an appropriate candidate for percutaneous ultrasound-guided breast cryoablation. Clopidogrel was held for five days according to institutional protocol. The procedure was performed with moderate sedation, local anesthesia, continuous cardiorespiratory monitoring, and real-time ultrasound guidance.

Technique

**Figure 3:**
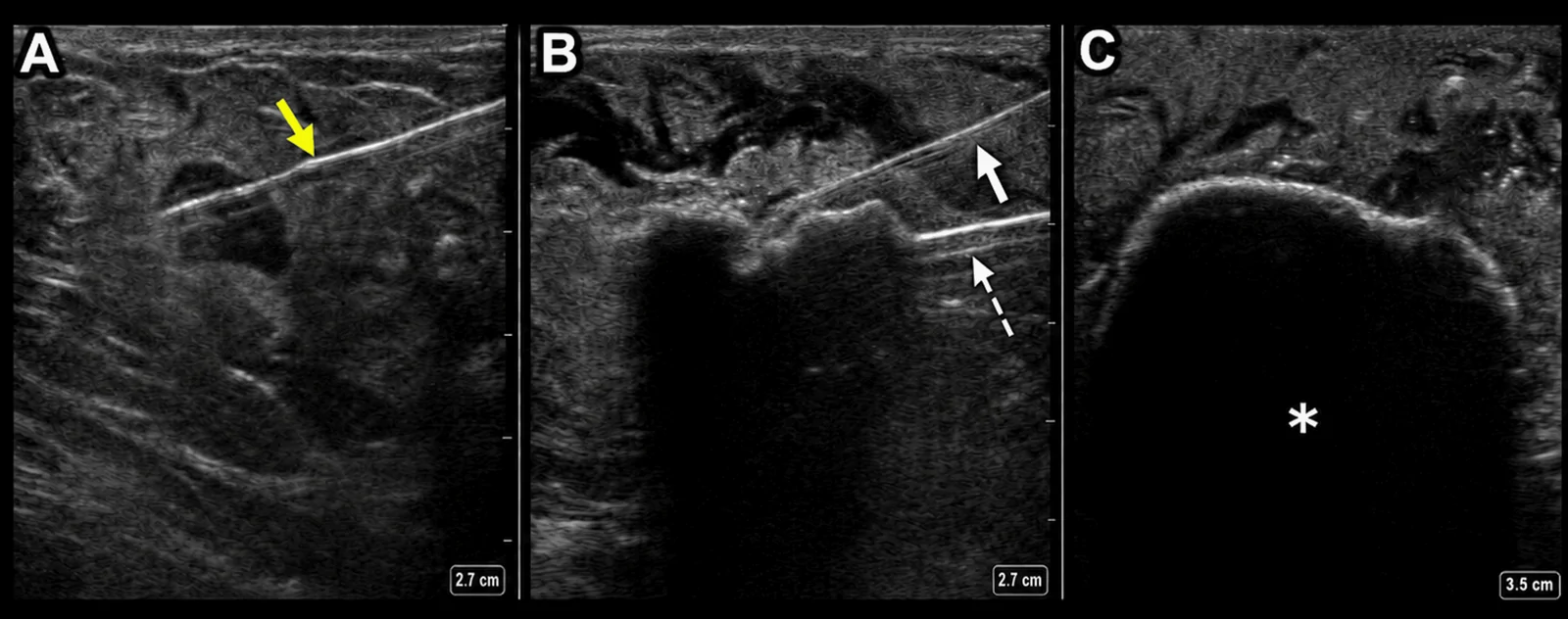
Intraprocedural images. (A) Cryoprobe (arrow) positioned through the biopsy site, centered on the HydroMark clip. (B) Hydrodissection needle (solid arrow) positioned superficial to the target, with the cryoprobe (dashed arrow) below it; the intervening saline creates a protective fluid plane between the anticipated ice ball and the overlying skin. (C) Echogenic leading edge of the ice ball (asterisk) during the freeze cycle, completely encompassing the clip-centered target.

The patient was positioned supine, and the right breast was prepared and draped using standard sterile technique (Figures 3A-3C). Ultrasound was used to identify the HydroMark clip corresponding to the previously biopsied lesion, and the overlying skin was marked.

Under continuous ultrasound guidance, a 17-gauge IceSeed 1.5 CX 90° cryoablation needle (Boston Scientific Corporation, Maple Grove, MN, USA) was advanced through the clip-centered biopsy site. The trajectory was planned to maximize target coverage while maintaining a safe distance from the skin. A separate 18-gauge spinal needle was positioned between the target and the skin for continuous hydrodissection with sterile saline, creating and maintaining a protective fluid plane throughout freezing [3,4]. Hydrodissection increased the skin-to-target distance from approximately 10 mm to approximately 20 mm, maintaining this fluid buffer throughout both freeze cycles.

A 10-minute freeze followed by a 5-minute passive thaw was performed under real-time ultrasound monitoring of ice-ball formation. Minor probe repositioning was then performed to optimize geometric coverage of the clip-centered target, followed by a second 10-minute freeze and 5-minute passive thaw. Hydrodissection was continued throughout both freeze cycles. The clip served as the primary landmark for target localization and probe positioning, whereas real-time ultrasound was used to monitor the expanding ice ball and confirm circumferential target coverage [3,5-7].

Ultrasound confirmed that the visible ice ball completely encompassed the clip-centered target while remaining separated from the skin by the hydrodissection plane. After probe removal, the treatment site appeared as an expected hypoechoic cryolesion. The visible ice ball measured approximately 2 cm in maximal diameter. Because the sonographic edge of the ice ball does not necessarily represent the boundary of lethal tissue injury, treatment adequacy was described by complete sonographic coverage rather than by an assumed fixed cytotoxic margin.

Hemostasis was achieved with manual pressure, and a sterile dressing was applied. The patient tolerated the procedure well, without immediate complications or evidence of injury to the overlying skin, chest wall, or adjacent structures.

Post-procedure follow-up

Follow-up was designed to evaluate early treatment response and detect suspicious residual or recurrent tumor. The first post-ablation assessment was performed at two months with clinical examination, targeted ultrasound, and contrast-enhanced breast MRI. Subsequent surveillance was planned at six-month intervals for the first two years and annually thereafter, with biopsy reserved for new nodular enhancement, interval growth, or another suspicious imaging finding [3,4].

**Figure 4:**
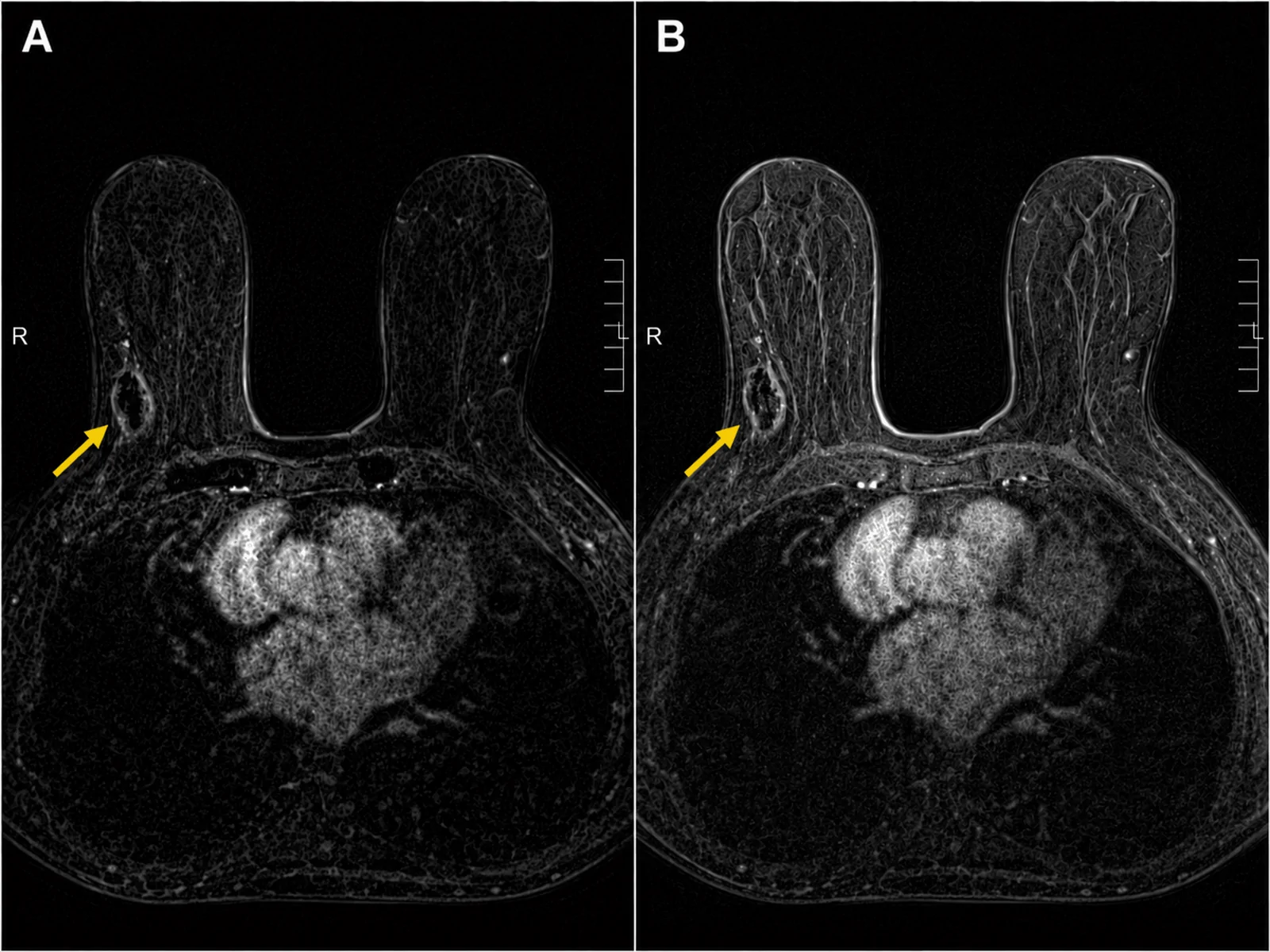
Two-month post-ablation MRI. (A) Precontrast fat-suppressed T1-weighted 3D gradient-echo image demonstrates the post-ablation cavity in the upper outer right breast. (B) Early postcontrast fat-suppressed T1-weighted 3D gradient-echo image demonstrates expected thin, smooth, circumferential rim enhancement of the ablation cavity without suspicious nodular or mass-like enhancement.

At two-month follow-up, clinical examination and targeted breast ultrasound demonstrated expected post-ablation changes without a suspicious residual mass. Contrast-enhanced breast MRI (Figures 4A-4B) demonstrated an ablation cavity measuring 2.9 × 1.9 × 1.4 cm (AP × TRV × CC) centered at the treated site, with expected treatment-related signal changes and peripheral enhancement, without suspicious nodular or mass-like enhancement to suggest residual viable tumor. The peripheral enhancement was thin, smooth, and circumferential, surrounding the ablation cavity in a uniform rim rather than forming a focal nodular, irregular, or eccentric mass-like focus. This pattern is consistent with the inflammatory and granulation-tissue response expected after thermal ablation and was distinguished from residual tumor, which typically manifests as nodular, irregular, or clumped enhancement along the cavity margin.

Following medical oncology evaluation, adjuvant letrozole was initiated because of the patient's postmenopausal, hormone receptor-positive disease. The intended duration of endocrine therapy will be determined by the treating oncologist according to tolerance and oncologic risk [8].

Longer-term clinical and imaging follow-up remains ongoing. The two-month evaluation establishes technical success and an encouraging early imaging response but is insufficient to demonstrate durable local tumor control or exclude late recurrence.

## Discussion

This case illustrates the potential role of image-guided percutaneous cryoablation as a local treatment option for carefully selected patients with small, biologically favorable IDC who are poor surgical candidates. Breast-conserving surgery remains the standard treatment for early-stage breast cancer, and cryoablation should therefore be considered within a multidisciplinary framework. In this patient, the procedure was completed under moderate sedation as an outpatient intervention, avoiding general anesthesia, surgical wound morbidity, and prolonged recovery. Published series have shown favorable tolerability and encouraging local control in selected elderly or medically fragile patients, although randomized comparisons with surgery remain unavailable [2,7,9].

Patient selection remains central to breast cryoablation. The patient's age, unifocal clinically node-negative tumor, Grade 1 histology, and ER-positive, PR-positive, HER2-negative phenotype were broadly consistent with the low-risk population studied in ICE3 [2]. However, ICE3 required a sonographically visible target throughout treatment, whereas this tumor lost conspicuity after biopsy and was localized by the clip; the patient's prior laryngeal and vulvar carcinomas and substantial comorbidity burden also place her outside the population enrolled in that registry. Beyond eligibility, ICE3 was a prospective single-arm study and did not establish oncologic equivalence to surgery. Similarly, ACOSOG Z1072 relied on subsequent surgical excision for pathologic confirmation, whereas the present case was assessed by imaging alone [1]. These distinctions are important when counseling patients and interpreting early treatment response.

The principal technical feature of this case was the use of a sonographically visible HydroMark clip to localize a 3-mm tumor that became poorly conspicuous after biopsy. The marker enabled accurate identification of the biopsy bed and probe positioning, while the ice ball itself was monitored independently with real-time ultrasound. Post-biopsy mammography confirmed that the clip remained centered within the mammographic target, supporting its use as a fiducial landmark. Nevertheless, a clip marks the biopsy site rather than microscopic tumor boundaries; complete treatment therefore requires ablation of an appropriate surrounding tissue volume rather than treatment of the marker alone.

Adjuvant endocrine therapy was selected because of the patient's postmenopausal, hormone receptor-positive disease. In the ICE3 cohort, patients receiving endocrine therapy had favorable local outcomes, although this observational association does not prove that endocrine therapy compensates for incomplete ablation [2]. Longitudinal surveillance with contrast-enhanced imaging and targeted ultrasound remains essential because early post-ablation edema, fat necrosis, hemorrhage, and peripheral inflammatory enhancement may overlap with suspicious findings [3,4]. New nodular or mass-like enhancement, interval enlargement, or another discordant finding should prompt tissue sampling.

This report has several limitations. First, the two-month follow-up interval is insufficient to assess durable local control. Second, treatment response was determined radiologically without surgical-pathologic confirmation. Third, because the lesion measured only approximately 3 mm and was sampled with multiple large-core passes, the entire imaging-visible tumor may have been removed at biopsy; residual microscopic disease could not be confirmed, and treatment of the clip-centered target volume was undertaken based on this possibility rather than on demonstrated residual disease. Fourth, the relationship between the clip and microscopic residual tumor could not be directly confirmed. Finally, observations from a single patient cannot establish comparative effectiveness relative to surgery.

## Conclusions

Percutaneous cryoablation continues to develop as a minimally invasive local option for carefully selected patients with small, biologically favorable breast cancers who are poor surgical candidates. A recurring obstacle in this setting is the accurate targeting of tumors that lose sonographic conspicuity after core-needle biopsy, with localization rather than ablation becoming the limiting step. This case illustrates that a biopsy clip can serve as a reliable sonographic landmark for target localization and probe positioning, allowing the procedure to proceed when the tumor is no longer clearly visible. Favorable early imaging cannot, however, confirm complete ablation or durable control, and a single case cannot demonstrate equivalence to surgery. Clip-guided localization may broaden the applicability of image-guided cryoablation for appropriately selected patients, though prospective studies with pathologic correlation and longer follow-up are needed before routine adoption.
